# The American Academy of Dental Surgery

**Published:** 1876-04

**Authors:** 


### ARTICLE IV.

### *The American Academy of Dental Surgery.*

A regular meeting of the Academy was held in New York, on Tuesday evening, February 29th.

Dr. George H. Perine, President in the chair. The chairman of the committee on Dentists in the Army and Navy being absent, no report was made.

The Treasurer, Dr. R. P Perry, presented his report. Dr. George E. Meigs, librarian, exhibited his report, showing quite an addition to the library and museum since his last statement. The corresponding secretary, Dr. E. S.

Howard, presented a number of letters received from Fellows in different parts of this country and Europe, which had been received since the last meeting.

Among the number read from fellows, was the following from Dr. John D. Miles, President of the Mississippi State State Dental Association:—

"Sickness in my family will prevent my compliance with your kind request to be with you, and read a paper on the 29th instant. This I much regret, for the reason that I should reap great practical benefit from the proceedings of so experienced a body of scientists and original thinkers, and although unable myself to give any learned disquisition on the many obstruse subjects in the range of dental science, yet I might hope to produce something from the repository of twenty years of dental experience, that might entertain, if not instruct the most erudite."

Dr. Geo. W. Field, corresponding Fellow, writes from London, England. He says:

"We of this side of the Atlantic, feel that it is 'carrying coals to New Castle' to send news (professionally) from the old to the new world. Of all branches of science and of all arts, there are doubtless none except our chosen specialty, which may not claim Europe for its headquarters, and here they attain their highest perfection, practically and theoretically. While we have a Harris of the past, and a Garretson of the present, illustrating rise and progress, we have no need to look to Europe farther than to keep well in mind that great effort is being put forth in England to outstrip all that has gone before, and if possible to leave all mediate competitors far behind. And I must add, that the strides in *theoretical knowledge* are wonderful and worthy of emulation, but the great utibility of practical ability, is almost totally ignored by the most zealous labors in the advancement of dental education. At the present time, efforts put forth have but one object in view—to elevate the dental profession in England by the only legitimate and possible means to the same level, and the same recognition professionally and socially with the medical profession. This is slowly but surely being accomplished; with such men as Tomes, Coles, Cartwright, Fox, and others, bending all their forces to the work, and seconded by the powerful moral influence of the support of the College of Surgeons, what may we not expect from the dental profession of England? I would say to American schools, 'go thou and do likewise,' not neglecting the practical manipulation branch in which you as operators have no peers.

This neglect on the part of our English co laborers, is but an oversight which their strong common sense will right itself in time, and then my friend unless the irrepressible Yankee looks well to his laurels, they will be found on this side of the Atlantic

So far the American dentist in Europe has borne a most enviable reputation. So the '*Dentiste American*,' on the Continent was '*carte blanche*' to a full and successful practice even to a man of no ability, and whose best trait was American, although a graduate from a barber shop, or a three months' apprenticeship in a dental laboratory. This army of impostors have done their work, and a revolution is the legitimate result. They are being put down by the people who have their eyes opened to the fraud and impositions, and their pockets closed. The harvest is about past, and now we must succeed here as in America, by dint of hard work and honest conscientious successful operations. Of this class there are but few now in Europe, but they are gradually taking the lead and receiving the recognition that they deserve. All honor to them for all temptations to fall in the old time worn groove of slack operations 'all for gold.'

I have in these few lines endeavored to give you, although in a very feeble manner, an outline of the present status of our profession in Europe; and I trust that the retrograde conditions of fellow operators and countrymen in Europe, and the progressive state of the English professor may prove a spur and give double incentives for active, earnest, energetic effort to our educators in the United States.

Let animosities, jealousies and envies be put aside, and all put their shoulders to the wheels of our car of improvement, taking 'Professional Progress' for their motto."

Our Berlin corresponding Fellow, writes:

"I am sure words would fail me should I attempt to express my pride and gratification as I mark the excelsior movements of our profession. It is through such efforts as your honorable body are making, and the convocations of our scientific colleagues throughout America and Europe that we are being felt as a power, and recognized among the medical fraternity and the scientific and cultured of every land as benefactors to the race, ranking second to none. Such should be our positon, for is not suffering humanity through all the horrors of oral diseases turning hopeful eyes to us? and is not the family dentist as great a boon as the family physician.

We have grave responsibilities; we are exciting the criticism of those who never dreamed of recognizing us as of their fraternity, but as our artistic and scientific skill betrays itself, not only in manipulations, but in the scientific researches, discussions, mechanical appliances and inventions for the advancement of our profession, the cordial grasp of recognition, and the still more flattering desire to learn of us, bring us into the conscious realization that our efforts are not in vain.

All honor to the American Academy of Dental Surgery, because it is one other heroic flag unfurled, significant of progress and strength.

I regret that I cannot be with you, but I am at work in my Berlin office, and when I tell you there are only eighty-nine dentists in this city of nearly one million inhabitants, I am sure you will say, 'stay where you are.' I wish I had time and the inspiration to write 'an article' for you, that I might exercise my new dignity as corresponding Fellow; but both of these all essential commodities fail me.

We had our Continental convention in August last, and are congratulating ourselves. But I read the dental news from 'home,' with something of the sense of longing that I used to have in China, when myself and partner were the only available representatives, and our discussions and experiments in office and laboratory constituted the dental clinic and dental convocation in the *orient ;* all the official honors of the time and occasion being vested in ourselves. How we read up the dental journals, and send for all the latest improvements, and emulate each other in delicate skill and careful efforts to excel, jealous lest half the world lying between us and our colleagues, should not keep even step with the onward march.

Something of that old yearning stirs me as I read your letter and you speak of your meetings, but the post of duty is here in this Capital City of Germany.

As a people, the Germans have no idea of our profession, and must be educated up to the proper appreciation of its importance. Some leading minds in the medical and surgical department have recently assured me of their pleasure and surprise at the wonderful developments in our line.

When secured as patients, I have never found a more appreciative and grateful class of people than among my practice here."

With best wishes for the prosperity of the Academy, I remain very truly,

Yours Fraternally,

WM. C. EASTLACKE, D. D. S.

Our corresponding Fellow, Dr. Geo. H. Chance, Portland, Oregon, says :—

"It is to me a source of gratification to know that while one is trying to do what he can for the elevation of the profession in this far western section of our country, his brethren east who are laboring under more favorable circumstances, can stop for a moment to send words of cheer and recognition to those who are engaged in 'Missionary Work.'

Such action will help very much to stimulate and strengthen the 'spinal column' of those of us who are trying to do what we can to advance the standard of professional excellence amongst us. It would afford me great pleasure to be with you did circumstances permit. I shall have to forego that pleasure for the present. Wishing every success and prosperity to the Academy, I will try and 'dot down' a few thoughts for your consideration on some future occasion."

An act to regulate the practice of dentistry in the State of Georgia, was received from Dr. Geo. Paterson, President of the Georgia State Dental Association.

A number of gentlemen were then elected active and corresponding Fellows of the Academy.

The subject of holding the July meeting in Philadelphia, (which will occur on the fourth Tuesday, the 25th,) was discussed, and it was finally agreed upon, and the corresponding secretary instructed to inform all Fellows at home and abroad of the fact. By the letters already received, it is thought that most of the States will be represented in this Congress. A goodly number of the Fellows residing in Europe, have already signified their intention to be present. A liberal invitation to the profession will be extended.

The President then made a few remarks. He desired to congratulate the Academy upon the good work it was doing. He said the Academy had recently given birth to its first child (association,) which had been christened "The New York Academy of Dentistry," a charter for which had been already obtained. He thought there was no reason why this Association should not aid in forwarding the work so well commenced by its parent. This the Doctor said was only the beginning of the work. Like organizations in other cities would be formed through and out of the American Academy, all of which would help restore dentistry to the parent tree,—medicine.

He trusted ere long that every medical college and hospital will have a special department for instruction of dental diseases as for those of the eye, throat or ear. Such he said is the end and aim of the "American Academy of Dental Surgery," and from the encouragement which we have already received, we are confident of our ability to succeed in carrying out our design. He said he could see no reason why dentistry should be separated from medicine, any more than surgery or any of the other specialities. He thought the time had come to restore this branch which had been so long lopped from the parent tree, and which experience shows should properly be restored to its original position. The paper of the evening, by John C. Storey, M. D., D. D. S., was then read and discussed by the Fellows, when the Academy adjourned to the fourth Tuesday in March.

---

